# Cannonballs in *Trichomonas vaginalis* Infection: Morphologic Evidence of Parasite-Associated Neutrophilic Aggregates

**DOI:** 10.3390/pathogens15060588

**Published:** 2026-05-29

**Authors:** Ruku Shinohara, Yukimi Misawa, Shuichi Mizuno, Saeka Honda, Koki Kikuchi, Rei Settsu, Yosuke Kato, Kaori Okayama, Mizue Oda, Mitsuaki Okodo

**Affiliations:** 1 Department of Medical Technology, Faculty of Health Sciences, Kyorin University, 5-4-1 Shimorenjaku, Mitaka-shi 181-8621, Tokyo, Japan; shinohara2111h@std.kyorin-u.ac.jp (R.S.); yukimi.misawa@yamatokai.or.jp (Y.M.); mizuno1911h@std.kyorin-u.ac.jp (S.M.); honda2421n@std.kyorin-u.ac.jp (S.H.); kikuchi2211h@std.kyorin-u.ac.jp (K.K.); rei-settsu@ks.kyorin-u.ac.jp (R.S.); yosuke-kato@ks.kyorin-u.ac.jp (Y.K.); 2 Department of Medical Technology and Sciences, School of Health Sciences, International University of Health and Welfare, 2600-1 Kitakanemaru, Otawara-shi 324-8501, Tochigi, Japan; okayaman0811@std.kyorin-u.ac.jp; 3 Department of Gynecology, Genki Plaza Medical Center for Health Care, 1-105 Jinbocho, Chiyoda-ku 101-0051, Tokyo, Japan; m-oda@genkiplaza.or.jp

**Keywords:** *Trichomonas vaginalis*, inflammation, cannonball, neutrophil, Papanicolaou smear

## Abstract

Cannonballs, compact aggregates of neutrophils observed in Papanicolaou (Pap) smears, are frequently associated with *Trichomonas vaginalis* (*T. vaginalis*) infection but are generally regarded as nonspecific inflammatory findings. To clarify their morphologic features, we analyzed cervicovaginal liquid-based cytology specimens from 29 cervicitis cases, including six positive for *T. vaginalis*. Cannonballs were evaluated using immunocytochemistry for *T. vaginalis* and cytokeratin, cell block analysis, and morphometric analysis, with negative cases as controls. All positive cases contained *T. vaginalis*-associated cannonballs, with a mean positivity rate of 57.4%. Parasites were intermingled with aggregated neutrophils, and cell block analysis demonstrated parasite-centered neutrophilic aggregates. Cytokeratin staining patterns differed morphologically between positive and negative cases. In addition, *T. vaginalis*-associated cannonballs were significantly smaller than adjacent squamous epithelial cells. These findings suggest that some cannonballs in trichomoniasis may represent parasite-associated neutrophilic structures and provide insight into host–parasite interactions in cervicovaginal inflammation.

## 1. Introduction

Cannonballs are compact aggregates of neutrophils occasionally observed in inflammatory Papanicolaou (Pap) smears [1,2,3,4,5,6,7,8] and considered nonspecific indicators, because they can be observed in infections with *Trichomonas vaginalis* (*T. vaginalis*) [1,5,6,8], *Chlamydia trachomatis* [4], and *Ureaplasma* sp. [3], as well as bacterial vaginosis [2,6,7]. Cannonballs have traditionally been considered aggregates of neutrophils that adhere to squamous epithelial cells, associated with phagocytic responses to pathogens on the epithelial surface [2]. Although frequent in *T. vaginalis* infections and often considered a useful background indicator, their cellular composition and biological significance remain poorly understood.

This brief report presents a series of *T. vaginalis*-positive cervicitis cases and proposes a revised interpretation of cannonballs based on immunocytochemical and morphometric findings, with direct relevance to routine gynecologic cytology.

## 2. Materials and Methods

This study was approved by the Ethics Committee on Human Research of Kyorin University (approval No. 2023-1-1, approval date: 13 May 2025) and conducted in accordance with the approved guidelines. SurePath™ liquid-based cytology (LBC) cell pellet samples (Becton Dickinson, Franklin Lakes, NJ, USA) from 29 cases of cervicitis showing numerous isolated or aggregated neutrophils on Pap smears were retrospectively selected from the archives of the Genki Plaza Medical Center for Health Care, Tokyo, Japan. Among them, six were positive for *T. vaginalis*, and 23 were negative. Ten Pap-stained slides were prepared from each LBC specimen. All slides were independently evaluated by two certified cytotechnologists for the presence of cannonballs and *T. vaginalis*. In cases of discrepant interpretations, the final diagnosis was determined by consensus discussion between the observers. In addition, residual cell pellets were subjected to polymerase chain reaction analysis for *T. vaginalis*; the results were in agreement with cytological findings. For semi-quantitative assessment, cytokeratin staining patterns in cannonballs were classified by the same two blinded observers as diffuse, focal/fragmented, weak, or negative. Discrepant interpretations were resolved by consensus discussion.

Then, cell blocks were prepared from the six *T. vaginalis*-positive cases according to the method described by Sakamoto et al. [9] and 4 μm sections obtained for histologic and immunocytochemical analysis.

Cannonballs were defined as aggregates of ≥5 tightly cohesive polymorphonuclear leukocytes. Cannonballs in Pap smears were photographed at ×10 magnification. Immunocytochemical staining for *T. vaginalis* was performed on both Pap smears and cell block sections using a mouse monoclonal anti-*T. vaginalis* antibody (1:20, clone A63A; Thermo Fisher Scientific, Waltham, MA, USA). To evaluate the presence of epithelial components within cannonballs, additional immunocytochemical staining for cytokeratin was performed using a ready-to-use polyclonal anti-cytokeratin antibody (Nichirei Biosciences Inc., Tokyo, Japan). Immunoreactivity was detected using an alkaline phosphatase-based system with Fast Red as chromogen. To evaluate the spatial relationship between neutrophils and *T. vaginalis*, cell block sections were additionally stained for neutrophils using a peroxidase-based method with diaminobenzidine as the chromogen.

Morphometric analysis of cannonballs and adjacent squamous epithelial cells was performed using CellSens Standard 1.16 (Olympus, Tokyo, Japan). Statistical analyses were conducted using the Mann–Whitney U test, with *p* < 0.05 considered significant. Differences in cytokeratin staining pattern distributions were analyzed using the chi-square test, and effect size was assessed using Cramer’s V.

## 3. Results

All six *T. vaginalis*-positive cases contained cannonballs harboring *T. vaginalis*, as demonstrated by immunocytochemical staining. A total of 799 cannonballs were identified in positive cases; 459 (57.4%) were immunopositive, with case-specific positivity rates of 33.5–100% (Table 1).

**Figure 1:**
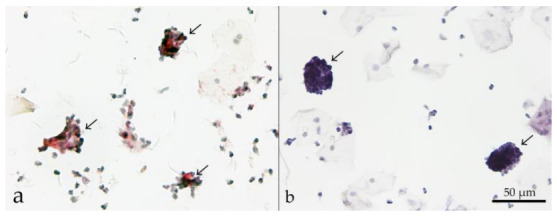
Immunocytochemical staining for *T. vaginalis* in cannonballs detected in Pap smears. (**a**) Cannonballs (arrow) in *T. vaginalis*-positive specimens showing multiple *T. vaginalis* organisms irregularly intermingled with aggregated neutrophils. (**b**) Cannonballs (arrow) in *T. vaginalis*-negative specimens showing no immunoreactivity for *T. vaginalis*. Magnification ×10.

In positive cannonballs, multiple *T. vaginalis* organisms were irregularly intermingled with aggregated neutrophils (Figure 1a). In contrast, the 1128 cannonballs identified in the 23 *T. vaginalis*-negative control cases were immunonegative (Figure 1b).

**Figure 2:**
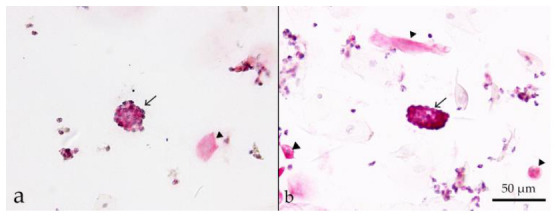
Cytokeratin immunocytochemical staining of cannonballs in cervicitis cases. (**a**) A cannonball (arrow) from a *T. vaginalis*-positive case showing focal/fragmented cytokeratin positivity. Adjacent squamous epithelial cells are indicated by arrowheads. (**b**) A cannonball (arrow) from a *T. vaginalis*-negative cervicitis case showing diffuse cytokeratin positivity. Adjacent squamous epithelial cells are indicated by arrowheads. Magnification ×10.

Additional cytokeratin immunostaining demonstrated epithelial positivity in both *T. vaginalis*-positive and -negative cannonballs [78/89 (87.6%) and 43/52 (82.7%), respectively]. However, cytokeratin staining patterns tended to differ between *T. vaginalis*-positive and negative cannonballs (χ^2^(3) = 7.669, *p* < 0.10), with a small-to-moderate effect size (Cramer’s V = 0.233) (Table 2). *T. vaginalis*-positive cannonballs more frequently exhibited focal/fragmented or weak staining patterns, whereas diffuse staining patterns were relatively more common in negative cannonballs. Representative findings are shown in Figure 2. *T. vaginalis*-positive cannonballs typically showed focal/fragmented cytokeratin staining (Figure 2a), whereas *T. vaginalis*-negative cervicitis cases frequently exhibited diffuse cytokeratin positivity (Figure 2b).

**Figure 3:**
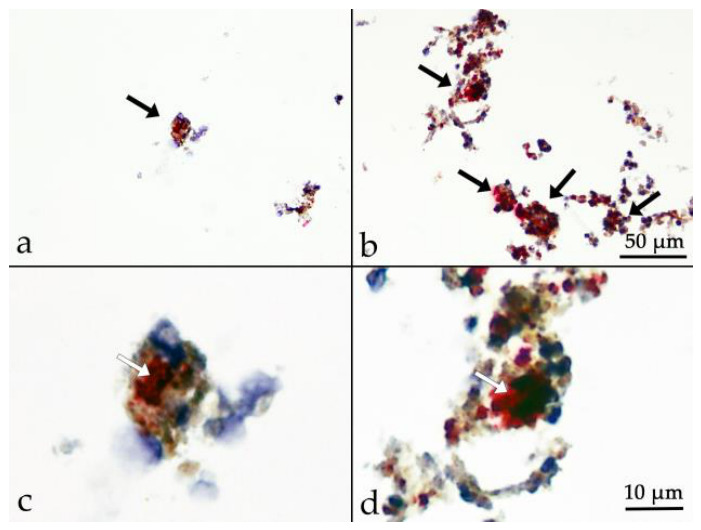
Cell block analysis of cannonballs in *Trichomonas vaginalis*-positive cervicitis cases. (**a**,**b**) Low-magnification views (×10) of cell block sections showing representative cannonballs (arrows). (**c**,**d**) High-magnification views (×40) of the corresponding cannonballs surrounded by neutrophils stained with peroxidase (brown). Open arrows indicate *T. vaginalis* organisms showing positive immunocytochemical staining (red signals) within the cannonballs.

Cell block sections from all six positive cases contained at least one cannonball in which *T. vaginalis* organisms were surrounded by neutrophils (Figure 3). Overall, 24 of 115 evaluated cannonballs (20.9%) in cell block sections were immunopositive for *T. vaginalis* (Table 1).

**Figure 4:**
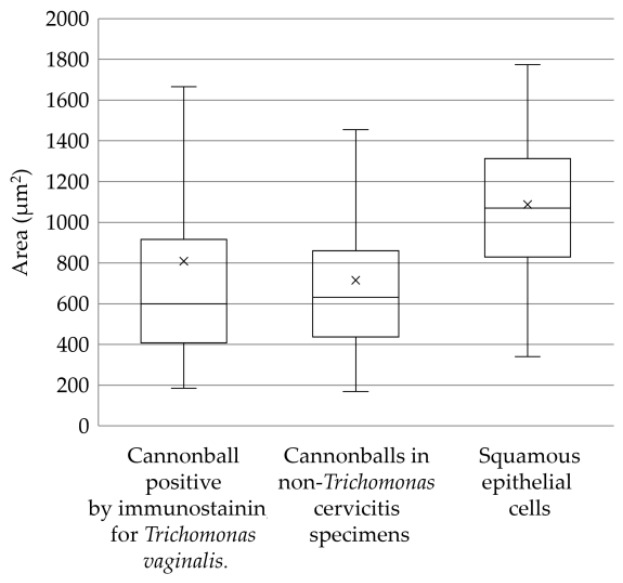
Box plots comparing the areas of *T. vaginalis*-immunopositive cannonballs, cannonballs from *T. vaginalis*-negative cervicitis cases, and adjacent squamous epithelial cells. Mann–Whitney U test, *p* < 0.01.

Morphometric analysis showed the mean areas (± SD) were 837 ± 831 μm^2^ for *T. vaginalis*-positive cannonballs, 715 ± 388 μm^2^ for negative cervicitis cannonballs, and 1199 ± 475 μm^2^ for squamous epithelial cells (Figure 4). *T. vaginalis*-positive cannonballs were significantly smaller than adjacent squamous epithelial cells (Mann–Whitney U test, *p* < 0.01). In contrast, no significant size difference was observed between cannonballs from positive and negative cases. Collectively, these findings suggest that cannonballs in trichomoniasis may represent parasite-associated neutrophil aggregates structurally different from nonspecific inflammatory cannonballs.

## 4. Discussion

The present results suggest that cannonballs in Pap smears from patients with *T. vaginalis* infection may represent parasite-associated neutrophilic aggregates rather than nonspecific inflammatory aggregates or neutrophils adherent to intact squamous epithelial cells.

This interpretation challenges the conventional view of cannonballs and provides a plausible explanation for their frequent association with trichomoniasis.

In cervicovaginal infections caused by coccoid or coccus-like bacteria, such as *Ureaplasma*, *Chlamydia*, and *Gardnerella vaginalis*, microorganisms typically adhere to squamous epithelial cells without markedly altering host cell morphology [10,11]. In such cases, neutrophils recruited by inflammatory mediators, including interleukin-8 [2,12], may cover epithelial cells in an effort to phagocytose surface-adherent bacteria, resulting in cannonballs seemingly containing intact squamous epithelium. In contrast, *T. vaginalis* adheres directly to epithelial cells and induces cellular damage and lysis [13]. In trichomoniasis, neutrophils mainly surround cellular debris and the parasite, rather than preserved intact epithelial cells, according to our immunocytochemical findings.

Experimental studies have shown that neutrophils kill *T. vaginalis* through trogocytosis rather than complete phagocytosis [14,15]. Given the parasite’s size and motility, complete engulfment by a single neutrophil is inefficient; therefore, multiple neutrophils may surround and interact with the parasite. This cooperative neutrophil response may contribute to the formation of cannonball-like structures observed in trichomoniasis. Such morphology may be suggestive of localized neutrophil–parasite interactions within the cervicovaginal microenvironment. Notably, immunopositivity was observed in 57.4% of cannonballs in Pap smears and 20.9% in cell block sections, suggesting heterogeneity in parasite-associated cannonball formation. Several factors may explain this heterogeneity, including sectioning effects in cell block preparations, technical limitations in immunocytochemical sensitivity or antigen preservation, and biologic variability in parasite-associated cannonball formation. Some cannonballs may therefore represent nonspecific neutrophilic aggregates rather than direct parasite-associated structures. Nevertheless, the absence of immunopositivity in cannonballs from *T. vaginalis*-negative cases and the frequent identification of *T. vaginalis* within cannonballs from positive cases support an association between cannonball formation and *T. vaginalis* infection.

Interestingly, although cytokeratin positivity was observed in cannonballs from both *T. vaginalis*-positive and -negative cases, the staining patterns differed morphologically. Cannonballs from negative cervicitis cases frequently showed diffuse cytokeratin positivity, whereas *T. vaginalis*-associated cannonballs more commonly exhibited disrupted staining patterns. Semi-quantitative assessment further supported this observation, as *T. vaginalis*-positive cannonballs more frequently exhibited focal/fragmented or weak cytokeratin staining patterns, whereas diffuse staining patterns were relatively more common in negative cannonballs. Although statistical significance was not reached, the observed distribution pattern was consistent with the morphologic impression that *T. vaginalis*-positive cannonballs often lacked intact epithelial cores. These findings suggest that epithelial material within trichomoniasis-associated cannonballs may largely represent disrupted epithelial remnants rather than preserved squamous epithelial cores. Despite these qualitative differences in cellular composition and cytokeratin staining patterns, cannonballs from *T. vaginalis*-positive and -negative cervicitis cases did not significantly differ in size. However, both types of cannonballs were significantly smaller than adjacent squamous epithelial cells. This finding may support the interpretation that cannonballs, particularly those in *T. vaginalis*-positive cases, do not contain preserved intact squamous epithelial cores. Instead, the epithelial material identified within these structures may represent fragmented or disrupted epithelial remnants. Nevertheless, area measurements alone cannot directly determine the structural integrity or cellular composition of cannonballs and should therefore be interpreted as supportive rather than definitive evidence.

Diagnostically, viewing cannonballs as neutrophil responses to parasites rather than epithelial adherence can help better interpret inflammation in cytology. The presence of numerous compact cannonballs lacking an identifiable epithelial core may prompt closer scrutiny for subtle parasitic forms or the use of techniques such as immunocytochemistry, particularly in LBC specimens, where organisms may be scarce or morphologically altered.

Several limitations should be acknowledged. First, the number of *T. vaginalis*-positive cases was relatively small, reflecting both the retrospective design of our study and the decreasing prevalence of trichomoniasis in screened populations. Second, although immunocytochemistry enabled *T. vaginalis* identification, it could not always distinguish between intact epithelial cells and severely disrupted cellular debris. Nevertheless, parasite-centered cannonballs were consistently observed in all positive cases, supporting the reproducibility of this morphologic pattern. Future studies evaluating neutrophil activation markers or oxidative stress-related molecules may help further clarify the biologic significance of cannonball formation in trichomoniasis. In addition, the current semi-quantitative assessment of cytokeratin staining patterns was based on morphologic classification by observers and may have been limited by the available sample size and staining variability. More robust quantitative approaches, such as digital image analysis, signal intensity quantification, or machine learning-based morphometric analysis, together with larger cohorts and standardized image-based quantification methods, may help further characterize subtle differences in epithelial distribution patterns within cannonballs and improve the reproducibility and objectivity of these observations.

## 5. Conclusions

Cannonballs in *T. vaginalis* infection may represent parasite-associated neutrophil aggregates reflecting host–parasite interaction. These findings provide additional insight into the morphologic features and potential diagnostic relevance of cannonballs in trichomoniasis.

## Figures and Tables

**Table 1:** Immunostaining for *Trichomonas vaginalis* in cannonballs detected in Pap smears and cell block sections.

	Case	*Trichomonas vaginalis* Positivity Rate of Immunostaining N Positive/Total (%)	
		Pap Smears	Cell Block Sections
*Trichomonas vaginalis* infection	1	244/403 (60.5)	8/34 (23.5)
	2	27/60 (45.0)	2/15 (13.3)
	3	31/37 (83.8)	3/7 (42.9)
	4	52/155 (33.5)	1/27 (3.7)
	5	21/60 (35.0)	1/11 (9.1)
	6	84/84 (100.0)	9/21 (42.9)
	Total	459/799 (57.4%)	24/115 (20.9)

Positive values indicate the number of immunopositive cannonballs relative to the total number of evaluated cannonballs.

**Table 2:** Semi-quantitative assessment of cytokeratin staining patterns in cannonballs.

Cytokeratin Staining Pattern	*Trichomonas vaginalis* -Positive Cannonballs N = 89	*Trichomonas vaginalis* -Negative Cannonballs N = 52
Diffuse	11 (12.4%)	15 (28.8%)
Focal or Fragmented	37 (41.6%)	16 (30.8%)
Weak	30 (33.7%)	12 (23.1%)
Negative	11 (12.4%)	9 (17.3%)

Data are presented as n (%).

## Data Availability

The data and materials supporting the findings of this study are available from the corresponding author upon reasonable request.

## Abbreviations

The following abbreviations are used in this manuscript:

LBC	Liquid-based cytology
Pap	Papanicolaou
